# Preconception health in current society: The PreconNet project

**DOI:** 10.18332/ejm/132714

**Published:** 2021-02-27

**Authors:** Petra Petročnik, Ana Polona Mivšek, Boštjan Žvanut, Patrik Pucer, Mirko Prosen

**Affiliations:** 1 Midwifery Department, Faculty of Health Sciences, University of Ljubljana, Ljubljana, Slovenia; 2 Department of Nursing, Faculty of Health Sciences, University of Primorska, Izola, Slovenia

**Keywords:** fertility, health, preconception, family planing

**Dear Editor,**

Statistics from different countries show a growing number of couples are postponing their decision to have children for several reasons^1^. These may lie in career development or other life goals that influence their family planning. For example, in Slovenia, the average age for first-time mothers in 1995 was 25 years, when in 2019 the average age was 29.6 years^2^. On the European level, the average age of women at the birth of their first child was 29.3 years in 2018^1^. However, postponing pregnancy may not only lead to pregnancy-related complications but also affects the couple’s ability to have children^3^. As studies show, the fecundity rate among women and men is declining^4^. In Slovenia, every sixth couple is having difficulties conceiving^5^, while on an international level it is estimated that every seventh couple is experiencing difficulties conceiving^6^. Apart from age, there are several other factors causing a decline in fertility rate among men and women. These include environmental factors such as endocrine disruptors, and lifestyle factors such as eating habits, sleep habits and physical activity. According to data from the World Health Organization^7^, the criteria for the normative quality of semen are getting lower with every year, which implies the reproductive ability of the human race is declining.

Considering this, there is a growing need to introduce preconception health from a different angle. In the past, family planning was merely oriented towards prevention of unwanted pregnancies and sexually transmitted infections. However, more and more authors started engaging with the topic of how to maintain fertility among men and women, which was also evident from the Lancet preconception health series^8^. Health professionals, especially midwives, have to start emphasizing the importance of the wider spectrum of preconception health and care by explaining to youth how to maintain their fertility and what factors may negatively impact their fertility.

For this reason, a number of experts from several European countries (including Finland, Austria, Belgium and Slovenia) started actively working on the Erasmus+ project, named ‘Preconception health of youth, bridging the gap in and through education’ (Supplementary file)^9^. The project is co-funded by the Erasmus+ programme of the European Union and aims to raise the level of fertility awareness throughout Europe. Within the project, we aim to implement preconception health issues in the curricula to strengthen preconception health among youth. The project also aims to accurately assess the situation regarding sexual and reproductive health in each partner country; and to raise the level of fertility awareness throughout Europe. However, the implications of the project will be useful for other European and non-European countries. The primary beneficiaries are participating educators and students, but also other healthcare professionals, parents, youth, children as well as the yet to be born^9^. Within the project, many publicly available tools are provided to help midwives and other health professionals in promoting preconception health and care. More information is available on the interactive Project Website: https://preco.tamk.fi/.

Midwives as health professionals in the field of reproduction will have a significant future role in promoting health and lifestyle activities to help youth maintain their fertility.
